# Shift Work Disorder in Nurses – Assessment, Prevalence and Related Health Problems

**DOI:** 10.1371/journal.pone.0033981

**Published:** 2012-04-02

**Authors:** Elisabeth Flo, Ståle Pallesen, Nils Magerøy, Bente Elisabeth Moen, Janne Grønli, Inger Hilde Nordhus, Bjørn Bjorvatn

**Affiliations:** 1 Norwegian Competence Center for Sleep Disorders, Haukeland University Hospital, Bergen, Norway; 2 Department of Psychosocial Science, University of Bergen, Bergen, Norway; 3 Occupational and Environmental Medicine, Uni Health, Bergen, Norway; 4 Department of Occupational Medicine, Haukeland University Hospital, Bergen, Norway; 5 Department of Public Health and Primary Health Care, University of Bergen, Bergen, Norway; 6 Department of Biological and Medical Psychology, University of Bergen, Bergen, Norway; 7 Department of Clinical Psychology, University of Bergen, Bergen, Norway; Institute of Psychiatry at the Federal University of Rio de Janeiro, Brazil

## Abstract

**Background:**

This study investigates the prevalence of symptoms of shift work disorder in a sample of nurses, and its association to individual, health and work variables.

**Methodology/Principal Findings:**

We investigated three different shift work disorder assessment procedures all based on current diagnostic criteria and employing symptom based questions. Crude and adjusted logistic regression analyses were performed with symptoms of shift work disorder as the dependent variable. Participants (n = 1968) reported age, gender, work schedule, commuting time, weekly work hours, children in household, number of nights and number of shifts separated by less than 11 hours worked the last year, use of bright light therapy, melatonin and sleep medication, and completed the Bergen Insomnia Scale, Epworth Sleepiness Scale, Global Sleep Assessment Questionnaire, Diurnal Scale, Revised Circadian Type Inventory, Dispositional Resilience (Hardiness) Scale – Revised, Fatigue Questionnaire, questions about alcohol and caffeine consumption, as well as the Hospital Anxiety and Depression Scale.

**Conclusions/Significance:**

Prevalence rates of symptoms of shift work disorder varied from 32.4–37.6% depending on the assessment method and from 4.8–44.3% depending on the work schedule. Associations were found between symptoms of shift work disorder and age, gender, circadian type, night work, number of shifts separated by less than 11 hours and number of nights worked the last year, insomnia and anxiety. The different assessment procedures yielded similar results (prevalence and logistic regression analyses). The prevalence of symptoms indicative of shift work disorder was high. We argue that three symptom-based questions used in the present study adequately assess shift work disorder in epidemiological studies.

## Introduction

In modern-day Western societies, round- the-clock performance is expected in many occupations. Census data show that a large segment of the workforce is employed on non-standard work schedules which may include shift work [1]. Such work schedules have been related to numerous health problems, among which are cardiovascular disease, digestive troubles, fatigue, cancer, depression/anxiety and, last but not least, sleep problems [2].

Individuals differ in terms of how they tolerate shift work, with effects on sleep and other health parameters varying correspondingly. Gender, age and personality traits such as diurnal type (morningness/eveningness, i.e. preference for going to bed and getting up early/late), circadian type (flexibility, i.e. ability to sleep and work at odd times, and languidity, i.e. lacking the ability to overcome drowsiness) and hardiness (resilience against environmental stressors) have been related to how, as well as the degree to which, individuals tolerate shift work [3].

Awareness of the mechanisms behind shift work related sleep problems could be essential regarding shift work scheduling, employment routines, clinical treatment as well as for employee selection [3]. However, it can be difficult to distinguish between the sleep issues related to shift work and those which are most likely unrelated [4]. Consequently, there is a need to conceptually differentiate between sleep problems associated with, and those bearing no relation to the work schedule [4], [5].

Shift work disorder (SWD) is a sleep disorder characterized by sleepiness and insomnia, which can be attributed to the person's work schedule. The diagnostic criteria for SWD, as defined by the American Academy of Sleep Medicine (AASM)'s International Classification of Sleep Disorders-2 (ICSD-2) [6], include: (i) complaints of insomnia or excessive sleepiness temporally associated with a recurring work schedule in which work hours overlap with the usual time for sleep, (ii) symptoms must be associated with the shift work schedule over the course of at least one month, (iii) sleep log or actigraphic monitoring for ≥7 days demonstrates circadian and sleep-time misalignment; (iv) sleep disturbance is not better explained by another sleep disorder, mental disorder, a medical or neurological disorder, medication use or substance use disorder [6]. As of today, few studies have explored SWD, and even fewer have systematically assessed the symptoms constituting the SWD diagnosis [4]. Furthermore, when symptoms such as insomnia and excessive sleepiness have been assessed, there have been variations in the instruments used and clinical cut-off values applied, resulting in differences in prevalence rates reported across studies [4]. Thus, there is a challenge in epidemiological research to reach an acceptable way to assess the symptoms of SWD in order to study its prevalence and associations with differential health problems and other relevant factors. Such research is needed to further understand how to better alleviate sleep problems related to work schedule. As of today, individual solutions have been emphasized (e.g. pharmacotherapy). A high prevalence of SWD symptoms may call for a focus on the systemic antecedents of work related sleep problems.

In the present study we assessed symptoms of SWD in a large sample of nurses working different shift schedules, aiming to:

1. Identify an acceptable procedure to assess SWD for epidemiologic purposes. We used three symptom-based questions based on the criteria for SWD according to the ICSD-2 [6], which have been described in a previous study [5]. If participants confirmed all three symptoms, this was considered indicative of SWD. In addition, we explored two other assessment procedures in order to study the impact these may have on the prevalence rate. These other procedures included fulfilling the criteria for insomnia and excessive sleepiness as well as excluding participants with other possible sleep disorders. If differences in prevalence were small between these procedures, this may indicate that the three symptom-based questions sufficiently assess SWD.
2. Study the relationship between symptoms of SWD and work schedule (e.g. day or night work, number of shifts separated by less than 11 hours off duty, and number of nights worked over the last 12 months), gender, age and health problems including insomnia, excessive sleepiness, fatigue, anxiety and depression; as well as: Commuting time, average number of hours worked per week, presence of children in the household, alcohol and caffeine consumption, sleeping aids and personality variables (circadian type, diurnal type and hardiness). We expected a positive relationship between symptoms of SWD and various health problems, which would highlight the impact of SWD as well as indicate that our SWD self-report measurement adequately discriminated between SWD-positive and SWD-negative subjects.

## Methods

### Ethical statement

This study was approved by the Regional Committee for Medical and Health Research Ethics, Health Region West (REK-Vest). Written informed consent was obtained.

### Procedure and participants

A sample of 5400 nurses working at least half-time was randomly selected from the Norwegian Nurses Organization's membership roll. This register comprises most of the Norwegian nurse population. The sample was organized into five equal strata based on number of years since the completion of basic nursing education (0–1 y, 1.1–3 y, 3.1–6 y, 6.1–9 y, and 9.1–12 y). During the winter 2008/2009, a questionnaire was administered by mail with a pre-paid envelope for return of the completed forms. Two reminders were sent, and the questionnaire was also made available online. Nurses were informed that provided participation; they would take part in a lottery rewarding 50 individuals with 500 NOK each. Among the 2 059 participants (response rate = 38.1%), 90.2% were females. A total of 69 participants were excluded as they worked less than half-time (equalling less than 17 h 45 min if working irregular hours, and 18 h and 45 min if only working permanent day shifts) or did not report their working position.

Demographic data were registered, including age, gender and whether or not respondents had children living at home. One-way commuting time was also assessed (0–15 min, 16–30 min, 31–45 min, 46–60 min, 61+ min). The nurses categorized their work schedule as either; i) permanent day shifts (7.5%) ii) permanent evening shifts (0.2%) iii) two-shift rotation comprising day and evening shifts (24.5%) iv) permanent night shifts (8.0%) v) three-shift rotation including day, evening and night shifts (56.9%), or vi) other work schedules including night work (2.8%). The category ii, “permanent evening shifts”, was omitted from analyses due to its low number of respondents (n = 4). Further, 18 participants were excluded as they did not report their work schedule. Consequently, the analyses for this study were based on a sample of 1968 nurses. The work schedule categories were dichotomized into daytime work (schedule i and iii, n = 631) or night work (schedule iv–vi, n = 1337).The nurses also reported their average number of hours worked per week, number of shifts separated by less than 11 hours off duty and the estimated number of nights worked during the last 12 months.

### Instruments

#### Shift work disorder

The present study employed questions previously developed and used specifically to assess/diagnose SWD in epidemiologic studies: (1) Do you experience difficulties with sleeping or excessive sleepiness? (yes/no), (2) Is the sleep or sleepiness problem related to a work schedule where you have to work when you would normally sleep? (yes/no), (3) Has this sleep or sleepiness problem related to your work schedule persisted for at least one month? (yes/no) [5]. These questions adhere to the symptoms/criteria listed in the ICSD-2 [6]. Respondents had to answer “yes” to all three questions in order to fulfil the criteria for SWD caseness. To ensure that the reported symptoms could be regarded as clinically severe, and that they were associated with the nurse's work schedule, we also investigated two other procedures for assessing SWD caseness, also based on the ICSD-2 criteria: one controlling for symptoms of various sleep disorders based on responses to the Global Sleep Assessment Questionnaire (GSAQ) [7] and one based on scores above clinical cut-offs on standardized self-report measures of insomnia [8], and sleepiness [9]. When using the first procedure, subjects were excluded from the SWD caseness group as assessed by the three-symptom questions if reporting considerable symptoms of other sleep disorders (see GSAQ section). For the second procedure, subjects had to confirm the three-symptom questions and in addition had to score above the clinical cut-off level on either insomnia or sleepiness measures, as described below, in order to fulfil the criteria for SWD caseness. As we did not perform clinical interviews, actigraphic measurements or administered sleep diaries, we did not *diagnose* the participants, but rather confirmed symptoms indicative of SWD.

#### Bergen Insomnia Scale (BIS)

The BIS is a self-administered insomnia scale, with symptom-related questions based on the American Psychiatric Association (APA)'s Diagnostic and Statistical Manual of Mental Disorders-IV- TR inclusion criteria for insomnia [10]. The scale has six items, which are scored along an eight-point scale indicating the number of days per week for which a specific symptom is experienced (0–7 days, total scores ranging from 0–42). Normative comparative data have been collected for the BIS, which has been validated using accredited subjective as well as polysomnographic data and found to provide sound psychometric properties [8]. Participants were categorized as insomniacs if scoring 3 or more on at least one of items 1–4, and 3 or more on at least one of items 5 and 6. In the current study, the Cronbach's alpha coefficient for the BIS was .83.

#### Epworth Sleepiness Scale (ESS)

The ESS constitutes eight items. Each item describes a specific situation for which respondents are asked to assess the likelihood of them falling asleep or dozing off on a scale ranging from 0 (would never doze off) to 3 (high chance of dozing off). The ESS score (clinical cut off ≥11) has been shown to allow for distinctions to be made between patients with various sleep disorders, and healthy subjects [9]. The ESS has shown high validity and reliability in numerous studies. A Norwegian version was used [11]. The Cronbach's alpha for the ESS was .74 in the present study.

#### Hospital Anxiety and Depression Scale (HADS)

The HADS is a self-assessment scale consisting of fourteen items, (scored on a four-point scale) measuring non-vegetative symptoms of anxiety (seven items) and depression (seven items) experienced during the last week [12]. The instrument has demonstrated acceptable reliability. A validated Norwegian version of the HADS was used in the present study [13], for which the Cronbach's alpha scores for both subscales were .82.

#### Global Sleep Assessment Questionnaire (GSAQ)

The GSAQ is a reliable and validated general sleep assessment tool which distinguishes between symptoms of different sleep disorders. We used four of the GSAQ questions as a screening tool for obstructive sleep apnoea, restless legs syndrome, periodic limb movement and parasomnias, respectively. The response alternatives are ‘never’, ‘sometimes’, ‘usually’, and ‘always’ [7]. The GSAQ was adapted to Norwegian by a standard translation-back-translation procedure. In the present study, subjects answering “always” to one or more of the four questions were excluded according to one of the SWD assessment procedures.

#### Fatigue Questionnaire

The 11-item Fatigue Questionnaire is a commonly used tool for measuring fatigue [14]. Items are scored on four-point Likert scale. The scale is divided into two dimensions: Physical Fatigue, based on the seven first items (range 0 to 21), and Mental Fatigue, based on the last four items (range 0 to 12). A Norwegian version of the questionnaire was used [15]. In the present study, the Cronbach's alpha was .89 for the Physical Fatigue scale and .84 for the Mental Fatigue scale.

#### Dispositional Resilience (Hardiness) Scale – Revised (DRS-15-R)

The 15 item hardiness scale measures three aspects of hardiness; commitment, control and challenge [16]. Items are scored on a scale ranging from 0 (“not true”) to 3 (“completely true”), yielding a total score ranging from 0 to 45. A validated Norwegian version was used [17]. The Cronbach's alpha for the DRS-15-R was .74 in the present study.

#### Diurnal Scale

The Diurnal Scale measures morningness, and has demonstrated high reliability between measurements [18]. The scale contains 7 items scored on a scale ranging from 1 to 4, which are then summarized giving a total score of 7–28. The scale was adapted to Norwegian by a standard translation-back-translation procedure. A high score indicates a preference for getting up early in the morning (morningness). In this study the Diurnal Scale had a Cronbach's alpha coefficient of .65.

#### Revised Circadian Type Inventory (rCTI)

The rCTI has been designed to assess circadian phase (flexibility, 5 items) as well as the amplitude of the circadian rhythm (languidity, 6 items) [19]. Items cover topics such as habits and preferences in relation to sleep and work schedules, and are answered on a 5-point scale. Flexibility (range 5 to 25) has been related to the capacity to adapt the sleep - wake cycle to unfamiliar patterns, and languidity (range 6 to 30) to the lack of ability to overcome sleepiness when sleep deprived. The scale was adapted to Norwegian by a standard translation-back-translation procedure. In the present study, languidity and flexibility had the respective Cronbach's alpha coefficients of .69 and. 80.

#### Short Form of the Alcohol Use Disorders Identification Test (AUDIT-C)

The AUDIT-C assesses alcohol consumption. The total score ranges from 0 to 12 [20]. A Norwegian version of the AUDIT-C was used. In this study, its Cronbach's alpha was .57.

#### Caffeine consumption

The nurses were asked the following question concerning caffeine consumption: “How many cups of coffee/tea/cola (with caffeine) do you usually drink daily?”

#### Use of sleep medications and bright light treatment

The nurses were asked the following questions concerning sleep medication and bright light treatment: “Have you during the past year used: i) sleep medication, ii) melatonin, iii) bright light treatment, and/or iv) non-prescription sleep medication. They were informed that they could tick off several alternatives if appropriate.

### Statistical Analyses

We used PASW version 18 for the statistical analyses. Descriptive data on the prevalence (categorical variables), means and standard deviations (continuous variables) for SWD-negative and SWD-positive participants were calculated for each of the three SWD assessment procedures.

Logistic regression analyses were conducted with SWD as the dependent variable (this was done for all three assessment procedures). Preliminary analyses were performed to exclude the possibility of collinearity. We included age, gender, night or day time work schedule, commuting time, average number of hours worked per week, presence of children in the household, number of night shifts worked over the last 12 months, number of shifts separated by less than 11 hours off duty, insomnia (not included in the analysis when the SWD assessment included insomnia as an additional criterion), sleepiness (not included in the analysis when the SWD assessment included sleepiness as an additional criterion), diurnal type, languidity, flexibility, hardiness, physical and mental fatigue, anxiety, depression, alcohol consumption, caffeine consumption, bright light therapy, melatonin use and sleep medication use (prescription and non-prescription) as predictor variables. All variables were first entered separately (crude analyses) and subsequently together in an adjusted analysis. Where the 95% confidence interval did not include 1.00, the odds ratios were considered statistically significant.

## Results

When using the three symptom-based questions, we found that a total of 37.6% of the nurses fulfilled the criteria for SWD caseness. The prevalence showed minor changes when using the two alternative assessment procedures (table 1).

**Table 1:** Prevalence of symptoms of Shift Work Disorder (SWD) within different work schedules, according to three assessment procedures.

Work schedule	SWD^1^		SWD+sleep disorders^2^		SWD+insomnia/sleepiness^3^	
	%	(n)	%	(n)	%	(n)
Day work only	6.2	(9)	4.8	(7)	5.5	(8)
Two shift rotation	28.9	(137)	27.6	(131)	24.7	(117)
Night work only	44.3	(70)	43.0	(68)	34.2	(53)
Three shift rotation	44.3	(488)	42.9	(472)	38.8	(425)
Other schedule with night work	40.7	(22)	40.7	(22)	37.7	(20)
TOTAL	37.6	(726)	36.2	(700)	32.4	(623)

1 SWD based on three symptom questions.

2 SWD based on three symptom-based questions, additionally excluding subjects answering “always” on symptoms of restless legs, sleep apnoea, periodic limb movements or parasomnias.

3 SWD based on three symptom-based questions, additionally using clinical cut-offs on Bergen Insomnia Scale and Epworth Sleepiness Scale as diagnostic criteria.

When excluding subjects potentially suffering from other sleep disorders, and when including the BIS and the ESS as SWD caseness criteria, the respective prevalence rates for the whole group of nurses were 36.2% and 32.4%.

When using the three symptom-based questions, 44.2% of the nurses working night shifts reported symptoms indicative of SWD, whereas 23.6% of nurses who did not work night shifts reported symptoms indicative of SWD. As shown in table 1, symptoms of SWD were found among 6.2% of the subjects working daytime only, compared to 44.3% of subjects working on a three-shift rotation. Out of nurses working on a two- shift rotation, 28.9% had symptoms indicative of SWD. Table 2 shows the means and prevalences of the different variables in the SWD-positive group compared to in the SWD-negative group. The other SWD assessments gave similar means and prevalence rates (data not shown).

**Table 2:** Means, standard deviations and percentages regarding work, health and personality variables, among participants with and without symptoms of shift work disorder (SWD)^1^.

	No SWD^1^		SWD^1^	
	Mean	(SD)	Mean	(SD)
Age	32.8	(8.1)	33.7	(8.3)
Number of nights last 12 months	22.3	(27.4)	32.0	(30.9)
Shifts separated by <11 h	30.4	(27.4)	38.1	(27.7)
Bergen Insomnia Scale total score	10.7	(7.3)	18.2	(7.8)
Epworth Sleepiness Scale	7.7	(3.4)	9.5	(3.9)
Depression total score	2.1	(2.5)	4.0	(3.1)
Anxiety total score	3.9	(3.2)	6.0	(3.6)
Hardiness total score	32.0	(4.3)	30.3	(4.8)
Mental fatigue	4.1	(1.5)	4.8	(1.8)
Physical fatigue	8.4	(3.0)	10.4	(3.3)
Alcohol Consumption total score	3.9	(1.8)	3.9	(1.7)
Flexibility subscale	12.3	(4.1)	11.8	(3.9)
Languidity subscale	20.0	(3.6)	21.7	(3.7)
Diurnal Scale	18.1	(3.2)	17.0	(3.5)
Caffeine consumption	3.0	(2.9)	3.1	(2.4)

	No SWD^1^		SWD^1^	
	%	(n)	%	(n)
Gender				
Male	54.8	(102)	45.2	(84)
Female	63.2	(1098)	36.8	(640)
Work Schedule				
Day work	76.4	(473)	23.6	(146)
Night work	55.8	(733)	44.2	(580)
Fraction of full position				
50–75%	27.8	(335)	30.3	(220)
76–90%	14.2	(171)	12.5	(91)
>90%	58.0	(700)	57.2	(415)
Commuting time				
0–15 min	44.7	(537)	40.0	(290)
16–30 min	38.4	(462)	41.0	(297)
31–45 min	12.0	(144)	14.3	(104)
46–60 min	3.8	(46)	3.0	(22)
60 min or more	1.1	(13)	1.7	(12)
Having children in household	52.1	(600)	48.0	(337)
Sleep medication	4.6	(56)	12.7	(92)
Melatonin	0.7	(9)	3.6	(26)
Bright light therapy	1.7	(20)	3.6	(26)
Prescription free sleep medication	2.4	(29)	8.8	(64)

1 SWD based on the three symptom questions.

Crude logistic regression analyses (table 3) showed a significant relation between SWD as assessed by the three symptom questions, and the number of nights worked over the last 12 months, work schedule (day or night), having less than 11 h off between shifts, insomnia, sleepiness, depressive and anxiety symptoms, hardiness, mental and physical fatigue, languidity, morningness, use of sleep medication, melatonin, and non-prescription sleep medication (p<.001), as well as age, flexibility, bright light therapy (p<.01) and gender (p<.05).

**Table 3:** Crude regression analyses^1^, with symptoms of Shift work disorder (SWD)^2^^,^ 3 ^,^ 4 as the dependent variable.

	SWD^2^		SWD+sleep disorders^3^		SWD+insomnia/sleepiness^4^	
	OR	(95% C.I.)	OR	(95% C.I.)	OR	(95% C.I.)
Age	**1.01**	(1.00–1.03)	**1.01**	(1.00–1.03)	1.01	(0.99–1.02)
Gender male	1.00		1.00		1.00	
Gender female	**0.71**	(0.52–0.96)	**0.73**	(0.54–0.99)	0.75	(0.54–1.00)
Day work schedule	1		1.00			
Night work schedule	**2.56**	(2.07–3.18)	**2.61**	(2.10–3.25)	**2.44**	(1.95–3.06)
Commuting time						
60 min or more	1.00		1.00		1.00	
46–60 min	0.59	(0.26–1.30)	0.77	(0.34–1.73)	0.78	(0.34–1.80)
31–45 min	0.70	(0.31–1.53)	0.93	(0.41–2.09)	0.90	(0.39–2.06)
16–30 min	0.78	(0.34–1.78)	1.01	(0.44–2.35)	1.02	(0.43–2.40)
0–15 min	0.52	(0.20–1.32)	0.58	(0.22–1.52)	0.74	(0.28–1.95)
Fraction of full position						
>90%	1.00		1.00		1.00	
75%–89%	1.11	(0.90–1.37)	1.14	(0.92–1.40)	0.98	(0.79–1.22)
50%–75%	0.90	(0.68–1.19)	0.88	(0.66–1.17)	0.82	(0.61–1.10)
Children in household	1.00		1.00		1.00	
No children in household	0.85	(0.70–1.02)	0.86	(0.69–1.01)	0.85	(0.70–1.03)
Night shifts 12 months	**1.01**	(1.01–1.01)	**1.01**	(1.01–1.02)	**1.01**	(1.01–1.01)
Shifts with less than 11 h between	**1.01**	(1.01–1.01)	**1.01**	(1.01–1.01)	**1.01**	(1.01–1.01)
Bergen Insomnia Scale total score	**1.13**	(1.12–1.15)	**1.12**	(1.11–1.14)		
Epworth Sleepiness Scale	**1.15**	(1.12–1.18)	**1.13**	(1.10–1.16)		
Diurnal Scale	**0.90**	(0.88–0.93)	**0.90**	(0.88–0.93)	**0.89**	(0.87–0.92)
Languidity	**1.14**	(1.11–1.17)	**1.14**	(1.11–1.17)	**1.16**	(1.13–1.20)
Flexibility	**0.97**	(0.94–0.99)	**0.97**	(0.95–1.00)	**0.96**	(0.93–0.98)
Hardiness	**0.92**	(0.90–0.94)	**0.92**	(0.90–0.94)	**0.92**	(0.90–0.94)
Physical fatigue	**1.23**	(1.19–1.27)	**1.12**	(1.18–1.25)	**1.26**	(1.21–1.30)
Mental fatigue	**1.32**	(1.24–1.40)	**1.13**	(1.12–1.40)	**1.37**	(1.29–1.47)
Anxiety total score	**1.20**	(1.16–1.23)	**1.18**	(1.18–1.23)	**1.21**	(1.18–1.25)
Depression total score	**1.26**	(1.21–1.30)	**1.24**	(1.20–1.29)	**1.28**	(1.23–1.32)
Alcohol Consumption score	1.01	(0.96–1.07)	1.01	(0.96–1.07)	1.03	(0.97–1.08)
Sleep medication - No	1.00		1.00		1.00	
Sleep medication - Yes	**2.98**	(2.10–4.12)	**2.72**	(1.94–3.83)	**3.50**	(2.48–4.91)
Melatonin - No	1.00		1.00		1.00	
Melatonin - Yes	**4.94**	(2.30–10.60)	**4.53**	(2.16–9.48)	**6.24**	(2.91–13.39)
Bright light therapy - No	1.00		1.00		1.00	
Bright light therapy -Yes	**2.20**	(1.22–4.00)	**1.83**	(1.02–3.30)	**2.33**	(1.28–4.25)
Prescription free medication - No	1.00		1.00		1.00	
Prescription free medication -Yes	**3.92**	(2.51–6.14)	**2.14**	(1.19–3.84)	**3.70**	(2.41–5.70)
Caffeine consumption	1.01	(0.08–1.04)	1.01	(0.97–1.04)	1.01	(0.98–1.05)

1 Significant differences indicated in **bold**.

2 Shift work disorder SWD based on three symptom questions.

3 SWD based on three symptom-based questions, additionally excluding subjects reporting to always experience symptoms of other sleep disorders.

4 SWD based on three symptom-based questions, additionally having to score above clinical cut off on either Bergen Insomnia Scale or Epworth Sleepiness Scale.

According to the adjusted analysis (table 4), symptoms indicative of SWD was associated with age, night work, the number of shifts separated by less than 11 hours of time off, number of nights worked over the last 12 months, languidity and insomnia, all significant at p<.001; as well as with anxiety symptoms (p<.05). These variables all showed a positive relationship with SWD-caseness in the adjusted analysis. Flexibility and gender (male coded 0 and female coded 1) remained negatively related to SWD in the adjusted analysis (p<.001 and p<.05, respectively).

**Table 4:** Adjusted logistic regression analyses^1^, with symptoms of Shift work disorder (SWD)^2^^,^ 3 ^,^ 4 as the dependent variable.

	SWD^2^		SWD+sleep disorders^3^		SWD+insomnia/sleepiness^4^	
	OR	(95% C.I.)	OR	(95% C.I.)	OR	(95% C.I.)
Age	**1.05**	(1.02–1.07)	**1.04**	(1.02–1.06)	**1.03**	(1.01–1.06)
Gender male	1.00		1.00		1.00	
Gender female	**0.57**	(0.36–0.92)	**0.62**	(0.39–0.99)	0.70	(0.44–1.10)
Day work schedule	1.00		1.00		1.00	
Night work schedule	**3.08**	(2.13–4.44)	**2.95**	(2.05–4.24)	**3.40**	(2.36–4.90)
Commuting time						
60 min or more	1.00		1.00		1.00	
46–60 min	0.50	(0.15–1.68)	0.67	(0.19–2.30)	1.03	(0.29–3.70)
31–45 min	0.60	(0.18–2.03)	0.83	(0.24–2.85)	1.29	(0.36–4.64)
16–30 min	0.55	(0.16–1.94)	0.77	(0.21–2.76)	1.19	(0.32–4.44)
0–15 min	0.47	(0.11–1.90)	0.47	(0.11–1.98)	0.99	(0.23–4.23)
Fraction of full position						
>90%	1.00		1.00		1.00	
75%–89%	1.28	(0.92–1.80)	1.33	(0.95–1.83)	0.91	(0.66–1.27)
50%–75%	1.07	(0.71–1.62)	1.08	(0.72–1.63)	0.94	(0.63–1.40)
Children in household	1.00		1.00		1.00	
No children in household	0.76	(0.55–1.05)	0.77	(0.56–1.06)	0.84	(0.62–1.14)
Night shifts 12 months	**1.01**	(1.00–1.02)	**1.01**	(1.00–1.02)	**1.01**	(1.00–1.01)
Shifts with less than 11 h between	**1.01**	(1.00–1.01)	**1.01**	(1.01–1.02)	**1.01**	(1.00–1.01)
Bergen Insomnia Scale total score	**1.12**	(1.09–1.14)	**1.11**	(1.08–1.13)		
Epworth Sleepiness Scale	1.03	(0.99–1.08)	1.01	(0.97–1.05)		
Diurnal Scale	0.97	(0.92–1.02)	0.97	(0.92–1.02)	**0.95**	(0.90–0.99)
Languidity	**1.10**	(1.05–1.15)	**1.09**	(1.04–1.15)	**1.11**	(1.05–1.16)
Flexibility	**0.92**	(0.88–0.96)	**0.92**	(0.89–0.96)	**0.92**	(0.88–0.95)
Hardiness	1.01	(0.98–1.05)	1.00	(0.97–1.04)	1.02	(0.98–1.05)
Physical fatigue	1.03	(0.97–1.09)	1.03	(0.97–1.09)	**1.12**	(1.06–1.18)
Mental fatigue	1.07	(0.97–1.18)	1.07	(0.97–1.18)	1.01	(0.92–1.11)
Anxiety total score	**1.07**	(1.01–1.12)	1.05	(0.99–1.11)	**1.13**	(1.07–1.18)
Depression total score	1.05	(0.98–1.12)	1.05	(0.98–1.12)	**1.10**	(1.03–1.18)
Alcohol Consumption score	0.98	(0.90–1.07)	0.98	(0.90–1.07)	0.99	(0.91–1.08)
Sleep medication - No	1.00		1.00		1.00	
Sleep medication - Yes	1.12	(0.62–2.02)	0.89	(0.50–1.58)	**1.84**	(1.07–3.18)
Melatonin - No	1.00		1.00		1.00	
Melatonin - Yes	3.18	(0.92–10.98)	2.74	(0.85–8.81)	**6.52**	(1.95–22.04)
Bright light therapy - No	1.00		1.00		1.00	
Bright light therapy - Yes	0.54	(0.22–1.31)	0.52	(0.21–1.27)	0.95	(0.41–2.22)
Prescription free medication - No	1.00		1.00		1.00	
Prescription free medication - Yes	1.87	(0.97–3.51)	1.69	(0.89–3.20)	**2.50**	(1.35–4.65)
Caffeine consumption	0.95	(0.95–1.01)	0.95	(0.90–1.01)	0.97	(0.91–1.03)

1 Significant differences indicated in **bold**.

2 Shift work disorder SWD based on three symptom questions.

3 SWD based on three symptom-based questions, additionally excluding subjects reporting to always experience symptoms of other sleep disorders.

4 SWD based on three symptom-based questions, additionally having to score above clinical cut off on either Bergen Insomnia Scale or Epworth Sleepiness Scale.

As shown in table 3 and table 4, the logistic regression analyses based on the different SWD assessments yielded quite similar results. When subjects were excluded from the SWD-positive group due to the presence of other sleep disorders (based on GSAQ), anxiety did not remain related to SWD caseness. In the analyses in which BIS and ESS were added as criteria, gender was no longer associated with SWD caseness in the adjusted analysis. However, morningness, physical fatigue, depression, use of over-the-counter and prescription sleep medication and melatonin were all positively related to SWD caseness across the three different assessments of the latter construct.

## Discussion

About one third of the nurses in our population showed symptoms indicative of SWD, with highest prevalence in schedules involving night shifts. We also found a positive relationship between the numbers of nights worked and SWD. However, out of the 726 who reported symptoms of SWD, 146 were not working night shifts. Hence, some non-night work schedules may also entail an increased risk of SWD.

The prevalence of SWD caseness was high for all three assessment procedures (37.6%, 36.2% and 32.4% depending on procedure). On the other side, it may be just as remarkable that about 60% of the nurses did not report sleep or sleepiness problems in relation to their work schedules.

Few studies have used the formal symptoms criteria of SWD [4]. One study, written by Drake and colleagues, found a SWD prevalence of 10.0%, in a community-based sample [21]. Drake and colleagues (2004) assessed sleepiness using ESS (cut off >13), and insomnia using symptom based questions. The prevalence was calculated as the difference in prevalence between shift and day workers. We would argue that this procedure yields a too conservative prevalence estimate. Before the subtraction between prevalence rates, shift work and night work prevalence rates were 26.1% and 32.1% respectively. This procedure does not acknowledge that day work may also lead to insomnia and sleepiness [22].

We have previously reported a SWD prevalence of 23.3% among oil rig workers [5]. This is also lower than in the present study although identical diagnostic criteria were used. However, nurses have, in contrast to offshore workers, social/familial commitments alongside their occupational duties.

With high SWD prevalence rates, one may consider whether the diagnosis attributes a systemic, environmentally caused problem (i.e. work schedule) to the individual level (i.e. a person not handling work). Adapting a view of SWD as a systemic issue may allow a transfer of focus from individual solutions (pharmacotherapy, part-time employment) to systemic solutions (limiting the number of nights shifts, introducing flexible shift schedule solutions).

According to the ICSD-2, SWD symptoms should not be *better* accounted for by other diagnoses/health issues which may affect sleep [6]. Thus, having other health issues or sleep disorders do not imply that one may not also suffer from SWD. In this study we investigated different assessment procedures for symptoms of SWD. These different procedures only changed the prevalence figures (see table 1) and correlates slightly. According to the ICSD, the diagnosis can usually be diagnosed by history [6]. The symptom questions specifically ask whether individuals experience these symptoms in relation to their work schedule. These are the same questions a clinician would ask in a clinical assessment. There is no absolute certainty in an epidemiologic study that these symptoms are not better explained by other disorders. Still, it is likely that by using the alternative procedures described above, we may exclude individuals actually suffering from SWD. For instance, a subject may have SWD and in addition suffer from restless legs. Based on this, and the fact that all analyses yielded similar results, we argue that our three symptom questions hold merit in an epidemiological context. The subsequent discussion will thus be based mainly on the results from our analyses based on this assessment method.

Age was positively associated with symptoms of SWD in the adjusted analysis. This is in line with other studies showing increased sleep difficulties with shift work after 40 to 50 years of age [3]. Advancing age has been related to a higher sensitivity to circadian phase misalignment [23]. Also, getting enough sleep during the day may be difficult, as sleep in general tends to be less restorative with age [23].

Gender was associated with symptoms of SWD, with females showing a lower risk. Some previous studies have also favoured females [3]. It should be noted that there was a low proportion of men in this study. Also, nursing is still a highly female dominated occupation, thus the males in our sample represent a rather selected group. We controlled for presence of children in household, which was unrelated to symptoms of SWD. This may have corrected for some social differences that could have favoured male shift workers. Because of similar work type/work schedule, the gender difference is less likely to be due to confounding work-related variables.

We found night work to be an important risk factor, also regarding number of nights worked over the past year. Such a dose-response relationship is important as it may indicate a need for an upper limit of nights worked per year. We also found a positive relationship between SWD and number of shifts separated by less than 11 hours, in both the crude and adjusted analyses, which is in line with earlier findings [22]. It should be noted that these odds ratios (tables 3 and 4) were reported for “days per year”. Hence, a person with 50 night shifts will have a 50% greater probability of having SWD compared to a worker with 0 night shifts during the last year.

The insomnia score showed a positive relationship with symptoms of SWD in both crude and adjusted analyses, while the sleepiness-score was unrelated to SWD in the adjusted analysis. Although one would expect scores on both scales to be significantly related to symptoms of SWD, other studies have also failed to find a relationship between shift work and excessive sleepiness [24], and between SWD and excessive sleepiness [5]. The ESS does not ask about propensity to sleep while at work, whereas the SWD questions pertain to insomnia and sleepiness in the work context. Also, changes in work performance and issues affecting sleepiness such as caffeine consumption are not addressed in the ESS. Work related sleep problems may thus be present without causing elevation of the ESS scores.

Symptoms of SWD was negatively related to flexibility and positively related to languidity in both the crude and adjusted analyses. This is in line with studies by Di Milla et al (2005), showing that high scores on flexibility and low scores on languidity were both associated with an ability to perform at unusual times of the day. Morningness was positively and hardiness negatively associated with symptoms of SWD in the crude analysis, but showed no relation to SWD in the adjusted analysis.

Although fatigue scores were significantly related to symptoms of SWD in the crude analyses, both mental and physical fatigue were unrelated to SWD in the adjusted analysis. Fatigue has previously been associated with shift work (not SWD in particular) [24]. Anxiety symptoms entailed an increased risk of SWD in both the crude and adjusted analyses, while depressive symptoms were no longer related to SWD in the adjusted analysis. Psychological distress has often been reported in relation to night work when assessed in terms of general measures of negative affect [25]. Taking both constellations of symptoms into account, gives insight into possible differences between them in relation to SWD. Nevertheless, depression remained significant in the adjusted analyses using the ESS and BIS as additional criteria.

Use of sleep medication (both prescribed and over-the-counter), melatonin and bright light therapy were not related to symptoms of SWD in the adjusted analyses. All these aids showed significant relationships with SWD in the crude analyses, and they (except for bright light therapy) remained significant in the adjusted analysis including insomnia/sleepiness as an additional criterion. Also, as shown in table 2, each of the sleeping aids had been used by more than twice as many in the SWD-positive group compared to the SWD-negative group. It is possible that the SWD assessment method using insomnia/sleepiness cut-offs included a larger fraction of participants with more generalized sleep/sleepiness problems. These participants could have been more likely to use such aids. However, only a small fraction of the sample as a whole used sleeping aids, and the associations were not significant in the adjusted analysis. Neither alcohol nor caffeine consumption showed any relationship to symptoms of SWD in our analyses.

### Strengths and limitations

In the present study we used standardized and well-validated instruments. Additionally, the study was based on a large and homogenous sample of workers, limiting the influence from possible confounding variables (i.e. different work load, environment, work schedule, etc.). On the other hand, this homogeneity makes generalization to other occupations more problematic. Inclusion of a high number of independent variables can affect the ability to accurately detect differences in the analysis. Nevertheless, we performed necessary preliminary analyses ensuring that issues such as multicollinearity could be ruled out.

The present study has a quite low response rate. The response rates in epidemiologic research have been decreasing the past decades [26]. In a review on response rates, Baruch (1999) recommends further investigation when response rates fall outside the range of 60%±20% (±1 standard deviation from the mean response rate found in the mentioned review) [26]. The response rate in the present study (38.1%) was not dramatically below this norm, but issues regarding response rates should nevertheless be kept in mind. It is not possible to exclude the possibility that those participating in the survey had more sleep concerns than the general nursing population. Nevertheless, nonparticipation is often associated with poorer health status, decreasing the risk that the prevalence of SWD-caseness was inflated [27]. In addition, our sample shared important characteristics found in the Norwegian nursing population as a whole (i.e. distribution of gender and mean weekly work hours) [28]. Still, the prevalence rates in the present study need to be interpreted with caution due to the low response rate.

As the present study was cross-sectional, it is problematic to conclude on causal directions.

In this large scale epidemiological study it was not feasible to perform clinical interviews, actigraphic assessment or to administer sleep diaries. Hence, we could not *diagnose* the participants; nonetheless, we established the presence of symptoms indicative of SWD by asking three specific questions adhering to the core criteria of SWD.

In conclusion, we suggest that our three symptom-based questions are sufficient for assessing SWD caseness in epidemiological studies. More than one third of the nurses in our sample reported symptoms consistent with SWD. The present study found significant associations between symptoms of SWD and gender, age, night work, number of nights worked, working shifts separated by less than 11 hours, languidity/flexibility, anxiety and insomnia in the adjusted analysis.
